# Supplementary material for: Comparative Study on In Vitro Fermentation Characteristics of the Polysaccharides Extracted from Bergamot and Fermented Bergamot
Source: Foods. 2023 Jul 28;12(15):2878. doi: 10.3390/foods12152878 (PMC10418462; doi:10.3390/foods12152878)
Supplement: Supplementary file 1 [file foods-12-02878-s001.zip › foods-2466394-supplementary.pdf]

Supplementary Materials

Figure S1 Gut microbiota was altered by polysaccharides after *in vitro* fermentation. **(A)** Rarefaction curves of Shannon indexes, α-diversity indices of microbial community: **(B)** Chao1 and Simpson and **(C)** classification of bacteria at the phylum level in fermentation broth from different treatments. The control group (no additional carbon source supplement); BP (bergamot polysaccharide supplement); MLP (mono-Laoxianghuang polysaccharide supplement); TLP (tri-Laoxianghuang polysaccharide supplement); PLP (penta-Laoxianghuang polysaccharide supplement).

Figure S2 The concentrations of short-chain fatty acids (SCFAs) among different groups during *in vitro* fermentation. The control group (no additional carbon source supplement); BP (bergamot polysaccharide supplement); MLP (mono-Laoxianghuang polysaccharide supplement); TLP (tri-Laoxianghuang polysaccharide supplement); PLP (penta-Laoxianghuang polysaccharide supplement).

Figure S3 Peak areas of the 20 vital metabolites with variable importance in the projection (VIP value in the top 20). *$p < 0.05$, **$p < 0.01$, ***$p < 0.001$ compared to the control group., by one-way ANOVA with Tukey's post hoc test were considered. The control group (no additional carbon source supplement); BP (bergamot polysaccharide supplement); MLP (mono-Laoxianghuang polysaccharide supplement); TLP (tri-Laoxianghuang polysaccharide supplement); PLP (penta-Laoxianghuang polysaccharide supplement).

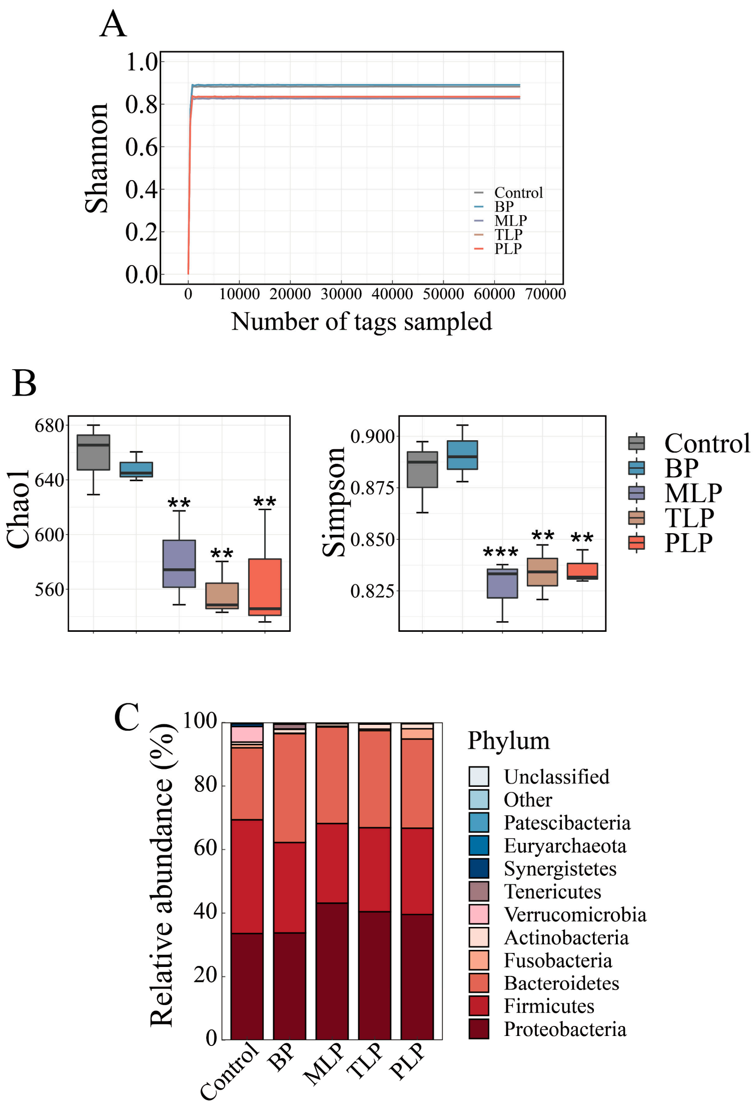

Figure S1

Gut microbiota was altered by polysaccharides after in vitro fermentation. **(A)** Rarefaction curves of Shannon indexes, α-diversity indices of microbial community: **(B)** Chao1 and Simpson and **(C)** classification of bacteria at the phylum level in fermentation broth from different treatments. The control group (no additional carbon source supplement); BP (bergamot polysaccharide supplement); MLP (mono-Laoxianghuang polysaccharide supplement); TLP (tri-Laoxianghuang polysaccharide supplement); PLP (penta-Laoxianghuang polysaccharide supplement). ** $p < 0.01$, *** $p < 0.001$

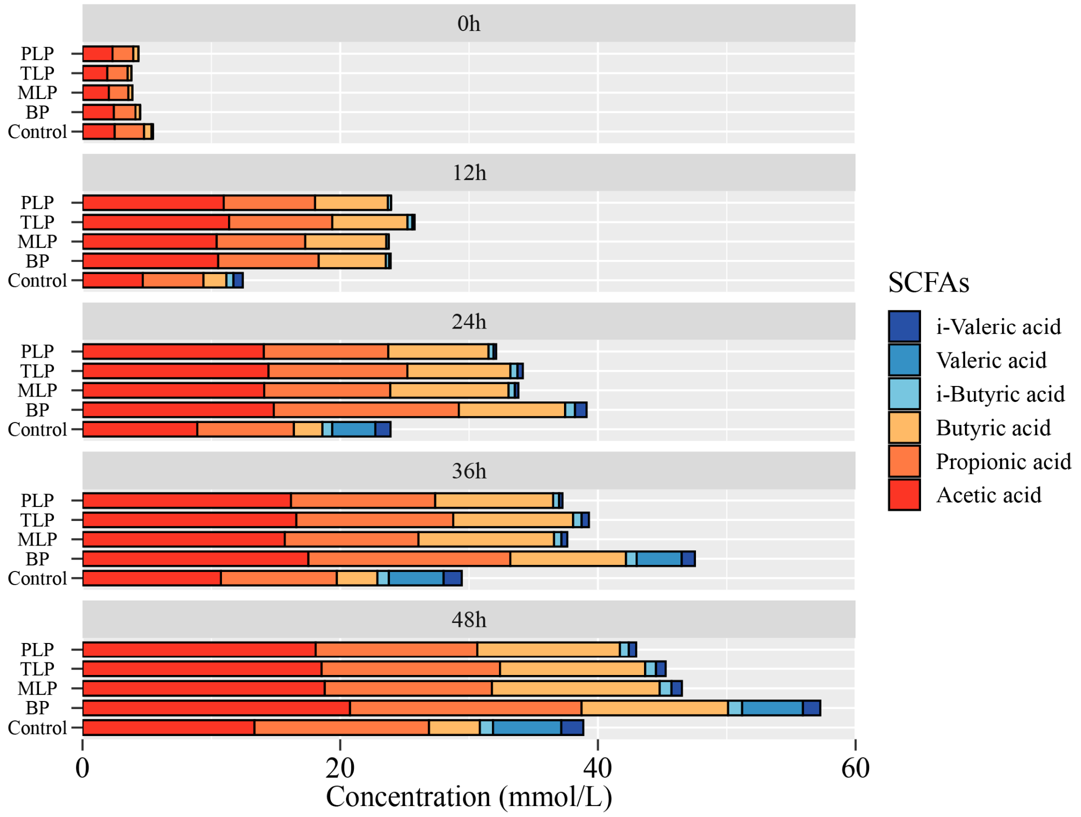

Figure S2

The concentrations of short-chain fatty acids (SCFAs) among different groups during *in vitro* fermentation. The control group (no additional carbon source supplement); BP (bergamot polysaccharide supplement); MLP (mono-Laoxianghuang polysaccharide supplement); TLP (tri-Laoxianghuang polysaccharide supplement); PLP (penta-Laoxianghuang polysaccharide supplement).

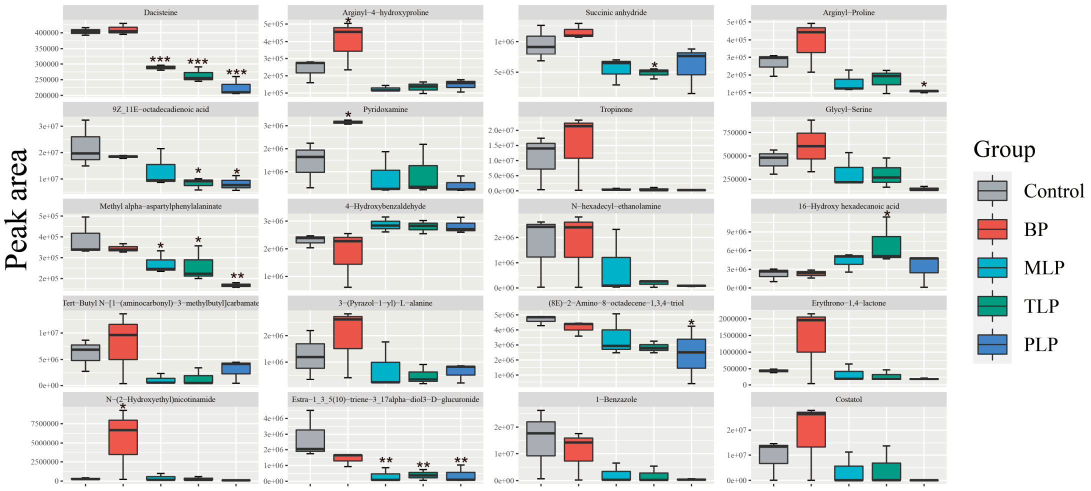

Figure S3

Peak areas of the 20 vital metabolites with variable importance in the projection (VIP value in the top 20). *$p < 0.05$, **$p < 0.01$, ***$p < 0.001$ compared to the control group., by one-way ANOVA with Tukey's post hoc test were considered. The control group (no additional carbon source supplement); BP (bergamot polysaccharide supplement); MLP (mono-Laoxianghuang polysaccharide supplement); TLP (tri-Laoxianghuang polysaccharide supplement); PLP (penta-Laoxianghuang polysaccharide supplement).
